# Morphological factors associated with progression of subaneurysmal aortas

**DOI:** 10.1093/bjs/znad030

**Published:** 2023-03-04

**Authors:** Knut Thorbjørnsen, Sverker Svensjö, Kevin Mani, Anders Wanhainen

**Affiliations:** Department of Surgical Sciences, Section of Vascular Surgery, Uppsala University, Uppsala, Sweden; Centre for Research and Development, Uppsala University, Region Gävleborg, Gävle, Sweden; Department of Surgery, Gävle County Hospital, Gävle, Sweden; Department of Surgical Sciences, Section of Vascular Surgery, Uppsala University, Uppsala, Sweden; Centre for Clinical Research, Uppsala University, Region Dalarna, Falun, Sweden; Department of Surgery, Falun County Hospital, Falun, Sweden; Department of Surgical Sciences, Section of Vascular Surgery, Uppsala University, Uppsala, Sweden; Department of Surgical Sciences, Section of Vascular Surgery, Uppsala University, Uppsala, Sweden; Department of Surgical and Perioperative Sciences, Surgery, Umeå University, Umeå, Sweden

## Abstract

**Background:**

The aim of this population-based cohort study was to assess the association between aortic morphological baseline factors in 65-year-old men with subaneurysmal aortic diameter (25–29 mm) and risk of later progression to abdominal aortic aneurysm (AAA) generally considered to be at a diameter for repair (at least 55 mm).

**Methods:**

Men with a screening-detected subaneurysmal aorta between 2006 and 2015 in mid-Sweden were re-examined using ultrasonography after 5 and 10 years. Cut-off values for baseline subaneurysmal aortic diameter, aortic size index, aortic height index, and relative aortic diameter (with respect to proximal aorta) were analysed using receiver operating characteristic (ROC) curves, and their associations with progression to AAA diameter at least 55 mm evaluated by means of Kaplan–Meier curves and a multivariable Cox proportional hazard analysis adjusted for traditional risk factors.

**Results:**

Some 941 men with a subaneurysmal aorta and median follow-up of 6.6 years were identified. The cumulative incidence of AAA diameter at least 55 mm at 10.5 years was 28.5 per cent for an aortic size index of 13.0 mm/m^2^ or more (representing 45.2 per cent of the population) *versus* 1.1 per cent for an aortic size index of less than 13.0 mm/m^2^ (HR 9.1, 95 per cent c.i. 3.62 to 22.85); 25.8 per cent for an aortic height index of at least 14.6 mm/m (58.0 per cent of the population) *versus* 2.0 per cent for an aortic height index of less than 14.6 mm/m (HR 5.2, 2.23 to 12.12); and 20.7 per cent for subaneurysmal aortic diameter 26 mm or greater (73.6 per cent of the population) *versus* 1.0 per cent for a diameter of less than 26 mm (HR 5.9, 1.84 to 18.95). Relative aortic diameter quotient (HR 1.2, 0.54 to 2.63) and difference (HR 1.3, 0.57 to 3.12) showed no association with development of AAA of 55 mm or greater.

**Conclusion:**

Baseline subaneurysmal aortic diameter, aortic size index, and aortic height index were all independently associated with progression to AAA at least 55 mm, with aortic size index as the strongest predictor, whereas relative aortic diameter was not. These morphological factors may be considered for stratification of follow-up at initial screening.

## Teaser

In this population-based screening cohort study, morphological baseline factors were characterized in individuals with a subaneurysmal aorta and risk of later progression to abdominal aortic aneurysm (AAA) of at least 55 mm. Aortic size index, aortic height index, and baseline aortic diameter were all independently associated with progression to clinically relevant AAA. Aortic size index was the best predictor; one-quarter of patients with an aortic size index of at least 13.0 developed an AAA of 55 mm or more by 10 years, compared with 1.2 per cent of patients with a value below 13.0. Some 47 per cent of the total cohort had an aortic size index of at least 13.0 and, based on this criterion, follow-up for subaneurysmal aorta could potentially be limited to this subgroup.

## Background

With the introduction of screening programmes for abdominal aortic aneurysm (AAA) and the increasing use of abdominal imaging^1,2^, more individuals are being identified with a subaneurysmal aorta (SAA; diameter 25–29 mm). There is, however, no general agreement on how these should be managed^3,4^. Data suggest that many SAAs eventually develop into AAAs, a significant proportion of which reach the threshold for repair within 10 years^5–7^. Consequently, the evidence to recommend re-examination of SAAs at 5–10 years after diagnosis is weak^8^. If it were possible to identify SAAs at risk of later reaching a diameter considered for repair (clinically relevant AAAs), a more selective follow-up routine could be established.

The aim of the present population-based screening cohort study was to assess the association between aortic morphological baseline factors and the risk of SAAs later developing into clinically relevant AAAs (diameter at least 55 mm) requiring surgical repair.

## Methods

The study was approved by the Ethics Committee of the Uppsala/Örebro Region (2006:112 and 2018/099). The Ethics Committee specified that informed consent was not required.

### Study setting

All men with SAAs detected by screening between 2006 and 2015 in four counties in mid-Sweden (Uppsala, Dalarna, Gävleborg, and Sörmland)^7^ were re-examined every 5 years. Men with an aortic diameter smaller than 25 mm at re-examination were discharged from the follow-up programme. Surveillance intervals for patients developing an AAA (aortic diameter 30 mm or more) were: 30–39 mm, every second year; 40–44 mm, annually; 45–49 mm, every 6 months; and 50–54 mm, every 3 months. Once the aortic diameter reached 55 mm or more, they were considered for AAA repair. Information on elective or acute AAA repair was retrieved from the patient records. Causes of death were retrieved from medical records linked to the National Population Registry.

Data on height, weight, risk factors, and family and medical history were obtained from standardized health questionnaires and medical records at the time of initial screening. Smoking status (current, former, and never smoker, smoking duration in smoking- and pack-years), history of AAA in first-degree relatives, coronary heart disease (defined as angina pectoris and/or myocardial infarction), hypertension, hyperlipidaemia, and diabetes mellitus (medical or dietary treatment) were recorded at the initial screening.

### Aortic size evaluation

Ultrasound measurements were made by registered nurses, specially trained in ultrasonography, or ultrasound technicians in certified vascular laboratories/units. The maximum anteroposterior diameter of the infrarenal aorta was measured according to the leading-edge-to-leading-edge (LELE) principle. The reproducibility of ultrasound measurements was evaluated in a previous study^9^ in which a small proportion of participants from the present cohort were included. In that study, LELE measurement was the most reproductible method of measuring the abdominal aorta. As a part of a regional protocol in three of the four counties, additional measurements of the suprarenal and/or adjacent proximal normal infrarenal aortic segment were taken.

The following morphological baseline factors were assessed in predicting progression to an AAA of 55 mm or larger: baseline SAA diameter^10,11^; aortic height index (AHI) based on height and aortic size index (ASI) based on body surface area (BSA)^12–15^; and relative aortic diameter quotient^16^ and relative aortic diameter difference between the maximum SAA diameter and that of the proximal adjacent aortic segment^17^ at the initial scan. BSA was calculated using the Du Bois formula: [0.20247 × (weight (kg)^0.425^) × (height (cm)^0.725^]^18^. A detailed description of these factors is provided in *Table 1*. For practical reasons, the best visible segment of the suprarenal, pararenal, and infrarenal aortic neck was considered as the proximal adjacent segment.

**Table 1:** Measurement methods and definitions

Morphological baseline factors	Aortic measurement methods
Baseline aortic diameter	Maximum AP SAA diameter (stratified by size in mm)
	Maximum AP SAA diameter (dichotomized by optimal cut-off value)
Aortic index based on height and BSA	AHI = maximum AP SAA diameter (mm)/height (m) (dichotomized by optimal cut-off value)
	ASI = maximum AP SAA diameter (mm)/BSA (m^2^) (dichotomized by optimal cut-off value)
Relative aortic diameter	Quotient = maximum AP SAA diameter (mm)/proximal adjacent aortic diameter (mm) (dichotomized by optimal cut-off value)
	Quotient = maximum AP SAA diameter (mm)/proximal adjacent aortic diameter (mm) ≥ 1.5
	Difference = maximum AP SAA diameter (mm) – proximal adjacent aortic diameter (mm) (dichotomized by optimal cut-off value)
	Difference = maximum AP SAA diameter (mm) – proximal adjacent aortic diameter (mm) ≥ 5.0 mm

AP, anteroposterior; SAA, subaneurysmal aorta; BSA, body surface area; AHI, aortic height index; ASI, aortic size index.

### Statistical analysis

Baseline characteristics are presented as median (i.q.r.) for continuous variables, and percentages with 95 per cent confidence intervals for dichotomous variables. Continuous variables were checked for normality using histograms. Proc MI in SAS^®^ was used for imputation of missing values. Logistic regression was used for dichotomous variables, and a linear regression model for continuous variables. The distribution of the imputed data was examined against the original data to control for eventual deviations in distributions. Cox proportional hazards regression univariable- (*Table 2*) and multivariable analyses (*Table 3*) were used to assess the association between morphological characteristics and AAA at least 55 mm adjusted for traditional AAA risk factors (hereditary, current smoking, coronary artery disease, hypertension, and hyperlipidaemia). Results are presented as HRs with 95 per cent confidence intervals. Log-log (survival) *versus* log (time) plots were used to test the proportional hazards assumptions of the Cox regression model. Progression to AAA at least 55 mm within 10 years stratified by morphological parameters was visualized in Kaplan–Meier (KM) curves. Time was calculated from the date of the initial scan to the first scan showing an aortic diameter of 55 mm or greater. Men with aortic diameters below this threshold were censored at the last recorded scan. Optimal cut-off values for the morphological variables to predict AAA at least 55 mm were determined based on receiver operating characteristic (ROC) curve analyses (*Figs S1a–S5b*). A threshold for sensitivity of ROC curves was set to 90 per cent or higher. Morphological variables and different relative aortic diameter AAA definitions were analysed by means of the log rank test. Correlation matrices were constructed for the strongest predictors (ASI, AHI, and baseline SAA diameter) (*Fig. S6*). Two-sided *P* < 0.050 was considered to indicate statistical significance. The statistical calculations were done using SPSS^®^ Statistics version 27.0 (IBM, Armonk, NY, USA) and SAS^®^ version 9.4 (SAS Institute, Cary, NC, USA).

## Results

**Fig. 1:**
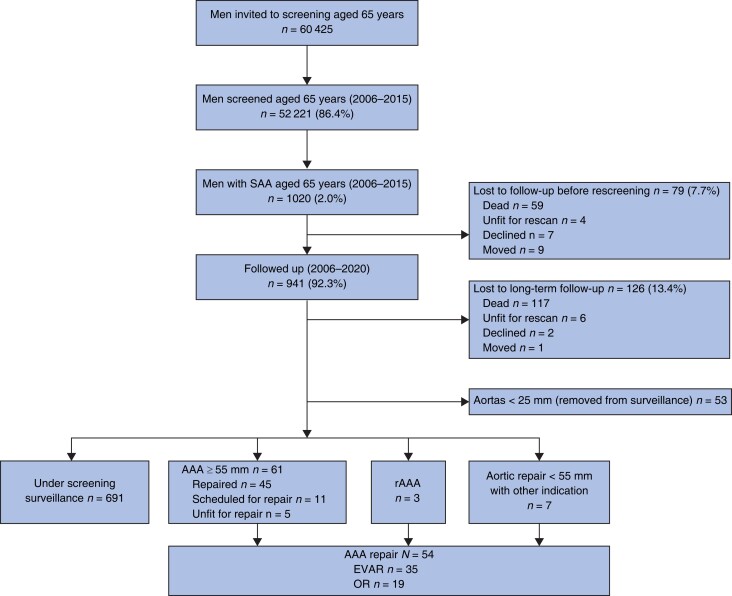
Flow chart of cohort of men with a subaneurysmal aorta measuring 25–29 mm at baseline ultrasound screening Causes of death of 176 men with a subaneurysmal aorta (SAA), regardless of follow-up time: malignancy 73; cardiac disease 40; pulmonary disease 15; stroke 10; trauma 6; sepsis 5; ruptured iliac aneurysm 1; other 13; unknown 13. AAA, abdominal aortic aneurysm; rAAA, ruptured AAA; EVAR, endovascular aortic repair; OR, open repair.

**Fig. 2:**
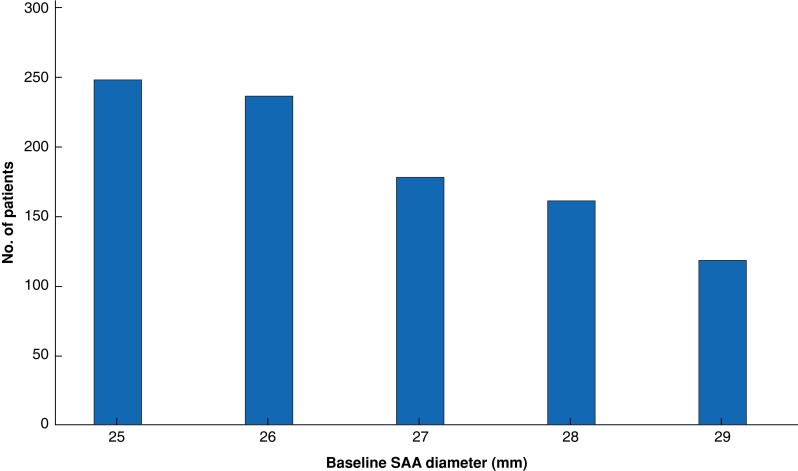
Subaneurysmal aortic diameters at initial ultrasound screening Data are shown for 941 men. SAA, subaneurysmal aorta.

Between 2006 and 2015, a total of 52 221 men were screened, of whom 1020 (2.0 (95 per cent c.i. 1.8 to 2.2) per cent) with an aortic diameter of 25–29 mm were identified. Some 941 (92.3 (90.7 to 93.9) per cent) had at least one valid follow-up ultrasound examination after the initial screening (*Fig. 1*). Information on height and weight was available for 735 men (78.1 (75.5 to 80.7) per cent) and a total of 524 (55.7 (52.5 to 58.9) per cent) had a valid scan of the proximal adjacent aortic segment. Median duration of follow-up until last scan after baseline screening was 6.6 (i.q.r. 5.0–9.4, range 0.5–14.1) years. The distribution of baseline SAA diameter at initial scan is shown in *Fig. 2*. The overall distribution of baseline characteristics is displayed in *Table 4*.

**Table 4:** Baseline characteristics

	No. of patients* (*n* = 941)	Missing (%)
Weight (kg), median (i.q.r.)	88 (80–97)	22.4
Height (cm), median (i.q.r.)	180 (175–184)	22.2
Body surface area (m^2^), median (i.q.r.)	2.07 (1.97–2.19)	22.4
BMI (kg/m^2^), median (i.q.r.)	27.4 (24.9–29.8)	22.4
**Baseline SAA diameter (mm), median (i.q.r.)**	26 (25–28)	0
ȃ≥ 26.0	693 (73.6)	0
**Aortic size index (mm/m^2^), median (i.q.r.)**	12.9 (12.0–13.8)	22.4
ȃ≥ 13.0	347 (47.2)	22.4
**Aortic height index (mm/m), median (i.q.r.)**	14.8 (14.1–15.6)	22.2
ȃ≥ 14.6	438 (59.5)	22.2
**Relative aortic diameter quotient, median (i.q.r.)**	13.9 (12.6–15.3)	44.3
ȃ≥ 50%	173 (33.1)	44.3
**Relative aortic diameter difference (mm), median (i.q.r.)**		44.3
ȃ≥ 5.0	449 (85.7)	44.3
**Smoking history**		
ȃSmoking-years, median (i.q.r.)	26 (0–40)	25.2
ȃPack-years, median (i.q.r.)	15.0 (0–33.6)	29.1
ȃCurrent smoker	276 (29.9)	1.8
ȃEver smoker	730 (79.0)	1.8
ȃNever smoker	194 (30.0)	1.8
First-degree relative with AAA	94 (10.8)	7.9
Coronary disease	257 (27.5)	0.5
Hypertension	538 (57.5)	0.5
Hyperlipidaemia	344 (36.9)	0.9
Diabetes mellitus	135 (14.4)	0.4

*Values are *n* (%) unless otherwise indicated. SAA, subaneurysmal aorta; AAA, abdominal aortic aneurysm.

Among the morphological factors, baseline SAA diameter, ASI, and AHI were all significantly associated with the development of AAA at least 55 mm at 10.5-year follow-up.

**Fig. 3:**
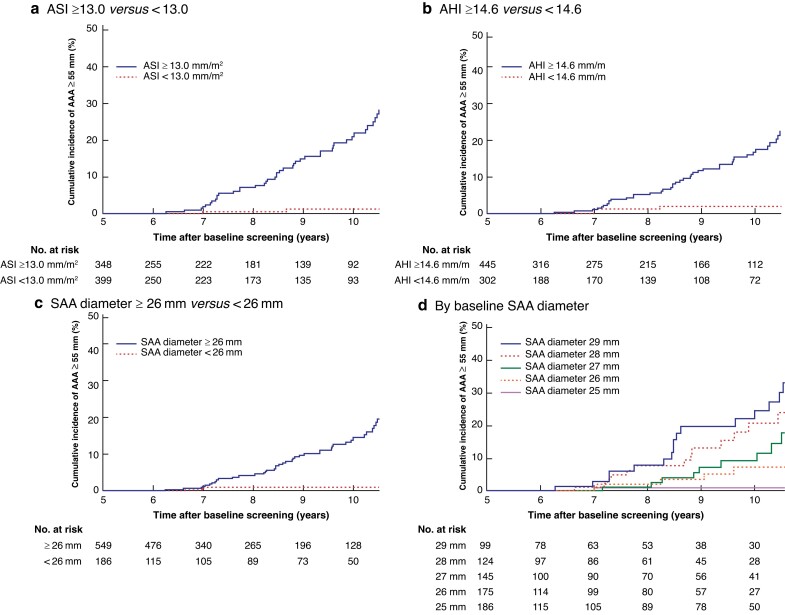
Kaplan–Meier estimates of cumulative incidence of baseline subaneurysmal aortic diameter, height, and body surface area with abdominal aortic aneurysm at least 55 mm as outcome variable **a** Aortic size index (AHI) 13.0 mm/m^2^ or more *versus* less than 13.0 mm/m^2^; **b** aortic height index (AHI) 14.6 mm/m or more *versus* less than 14.6 mm/m; **c** subaneurysmal aorta (SAA) (anteroposterior) diameter 26 mm or more *versus* less than 26 mm; and **d** SAA (anteroposterior) diameter stratified by size in millimetres.

The KM-estimated cumulative incidence of progression to AAA at least 55 mm at 10.5 years’ follow-up among men with an ASI of 13.0 mm/m^2^ or greater was 28.5 (s.e. 4.0) per cent *versus* 1.1 (0.8) per cent in men with an ASI of less than 13.0 mm/m^2^ (*P* < 0.001). The proportion of men with an ASI of 13.0 mm/m^2^ or higher at baseline was 45.2 (95 per cent c.i. 42 to 48) per cent (*Fig. 3a*).

The KM-estimated cumulative incidence of development of AAA at least 55 mm within 10 years in men with an AHI of 14.6 mm/m or more was 25.8 (s.e. 3.8) per cent *versus* 2.0 (1.1) per cent among in men with an AHI below 14.6 mm/m (*P* < 0.001). The proportion of men with an AHI of 14.6 mm/m or higher at baseline screening was 58.0 (95 per cent c.i. 55 to 61) per cent (*Fig. 3b*).

The 10-year KM-estimated cumulative incidence of men with an AAA reaching at least 55 mm with a baseline SAA diameter of 26 mm or higher was 20.7 (s.e. 3.1) per cent *versus* 1.0 (0.9) per cent of those with a baseline SAA diameter of less than 26 mm (*P* < 0.001). The proportion of men with an SAA diameter of 26 mm or greater at baseline was 73.6 (95 per cent c.i. 71 to 77) per cent (*Fig. 3c*).

After the 10-year follow-up scan, the KM-estimated incidence of AAA at least 55 mm was 1.0 (s.e. 0.9) per cent for men with a baseline SAA diameter of 25 mm, 7.3 (3.3) per cent for 26 mm, 17.7 (5.8) per cent for 27 mm, 24.4 (6.9) per cent for 28 mm, and 33.0 (7.1) per cent for 29 mm (*Fig. 3d*).

In a multivariable Cox proportional hazards regression model adjusted for traditional AAA risk factors (current smoking, heredity, cardiovascular disease), an ASI of 13.0 or more had an HR of 9.1 (95 per cent c.i. 4 to 23) (*P* < 0.001) for development of a clinically significant AAA; the HR for baseline SAA diameter 26 mm or greater was 5.9 (2 to 19) (*P* = 0.003) and that for AHI 4.6 or higher was 5.2 (2 to 12) (*P* < 0.001).

**Table 2:** Univariable Cox regression analyses for outcome abdominal aortic aneurysm at least 55 mm

	HR	*P*
Body surface area (m^2^)*	0.2 (0.42, 0.97)	0.045
BMI (kg/m^2^)*	1.0 (0.90, 1.03)	0.280
Baseline SAA diameter (mm)*	1.6 (1.29, 1.91)	<0.001
Baseline SAA diameter ≥ 26.0 mm	6.1 (1.91, 19.44)	0.002
Aortic size index (mm/m^2^)*	1.6 (1.26, 1.78)	<0.001
Aortic size index ≥ 13.0	9.0 (3.61, 22.61)	<0.001
Aortic height index (mm/m)*	1.8 (1.38, 2.26)	<0.001
Aortic height index ≥ 14.6	5.1 (2.21, 11.96)	<0.001
Relative aortic diameter quotient ≥ 1.2 (mm/mm)	1.2 (0.54, 2.63)	0.667
Relative aortic diameter quotient ≥ 50%	0.9 (0.52, 1.49)	0.641
Relative aortic diameter difference ≥ 5 mm	1.3 (0.57, 3.12)	0.506
Smoking-years*	1.0 (0.99, 1.05)	0.005
Pack-years*	1.0 (0.99, 1.06)	0.025
Current smoker	1.6 (0.95, 2.62)	0.081
Ever smoker	1.4 (0.67, 2.76)	0.395
Never smoker	0.7 (0.36, 1.49)	0.395
First-degree relative with AAA	1.9 (1.02, 3.54)	0.043
Coronary disease	1.1 (0.65, 2.01)	0.643
Hypertension	0.9 (0.52, 1.45)	0.593
Hyperlipidaemia	1.1 (0.63, 1.77)	0.830
Diabetes mellitus	0.9 (0.39, 2.13)	0.831

Values in parentheses are 95% confidence intervals. *HRs are shown per unit increase. SAA, subaneurysmal aorta; AAA, abdominal aortic aneurysm.

**Table 3:** Cox multivariable regression model for outcome abdominal aortic aneurysm at least 55 mm)

Factor	HR	*P*
Baseline SAA diameter ≥26.0 mm*	5.9 (1.84, 18.95)	0.003
Aortic height index ≥14.6*	5.2 (2.23, 12.12)	<0.001
Aortic size index ≥13.0*	9.1 (3.62, 22.85)	<0.001
Current smoker*	1.4 (0.84, 2.39)	0.191
Smoking-years*†	1.0 (1.0, 1.05)	0.026
First-degree relative with AAA	2.0 (1.06, 3.75)	0.032
Coronary disease	1.1 (0.66, 2.13)	0.683
Hypertension	1.0 (0.56, 1.74)	0.989
Hyperlipidaemia	1.1 (0.59, 1.94)	0.813

Values in parentheses are 95% confidence intervals. *Morphological variables (baseline subaneurysmal aorta (SAA) diameter, aortic height index, and aortic size index) and smoking variables were entered into the analysis separately. †HRs are shown per unit increase. AAA, abdominal aortic aneurysm.

**Fig. 4:**
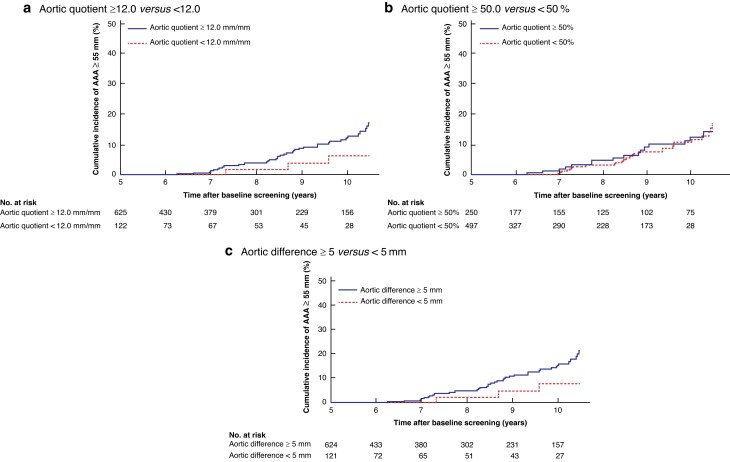
Kaplan–Meier estimates of cumulative incidence of relative subaneurysmal aortic diameter to proximal aorta at baseline with abdominal aortic aneurysm at least 55 mm as outcome variable **a** Relative aortic diameter quotient 12.0 or more *versus* less than 12.0; **b** relative aortic diameter quotient 50% or more *versus* less than 50%; and **c** relative aortic diameter difference 5.0 mm or more *versus* less 5.0 mm.

In contrast, no association was seen between progression to AAA at least 55 mm and the relative aortic diameter quotient ≥ 1.2 mm/mm (HR 1.2, 0.54 to 2.63; *P* = 0.667) or relative aortic diameter difference ≥ 5 mm (HR 1.3, 0.57 to 3.12; *P* = 0.506) (*Fig. 4*). Among traditional AAA risk factors, having a first-degree relative with an AAA (HR 2.0, 1.06 to 3.75; *P* = 0.032) and smoking-years (HR 1.0, 1.0 to 1.05) were found to be significant predictors of progression to AAA at least 55 mm. Pack-years (HR 0.99, 1.06 to 1.1; *P* = 0.025) was found to be significant, and current smoker (HR 1.6, 0.95 to 2.62; *P* = 0.081) showed a trend towards a significant difference in the Cox regression univariable analysis.

A total of 61 men (6.5 (95 per cent c.i. 5 to 8) per cent) with a screening-detected SAA reached the threshold diameter, AAA at least 55 mm, of whom 56 (92 per cent) were eligible for aneurysm repair. During the study interval, 45 (74 per cent) of these had undergone AAA repair and 11 (18.0 per cent) were awaiting a scheduled repair, whereas 5 men (8.2 per cent) were considered unfit for surgical treatment.

Three men were admitted with ruptured AAAs (rAAAs), all of whom underwent open surgical repair and survived. One ruptured 37-mm mycotic aneurysm appeared 4.5 years after the initial screening. The remaining two ruptures occurred 7 years after baseline screening; one man presented with a 56-mm rAAA 5 months after the last rescan with a measurement of 49 mm, and one presented with 47-mm saccular aneurysm associated with plaque rupture.

During the study interval (2006–2020), 176 men (17.3 per cent) died while under surveillance. No known AAA repair-related or rAAA-related deaths occurred; however, one man died from an undetected ruptured iliac aneurysm 5.5 years after baseline screening. Causes of death are noted in *Fig. 1*.

## Discussion

In the present study of a large cohort of men with screening-detected SAAs and long-term follow-up, morphological characteristics such as SAA diameter 26 mm or more, ASI 13.0 or higher, and AHI 14.6 or greater were strongly associated with an increased risk of progression to large (clinically relevant) AAAs. These factors could therefore serve as baseline predictors that can differentiate the population at risk of developing clinically relevant AAAs, and the need for follow-up (or not) of patients with screening-detected SAAs. Measures of abdominal aortic size (ASI, AHI, and baseline SAA diameter) were all valuable predictors of progression to AAA at least 55 mm; ASI was the best predictor with an up to nine-fold risk of progression to AAA with a diameter of 55 mm or more, and with less than half of the population at risk. Based on these findings, a more selective follow-up routine could be established.

In a recently published study^7^, it was shown that SAA developing into AAA of 30 mm or greater after 5 years of follow-up could serve as a predictor for later development of AAA at least 55 mm. Among those with an SAA that did not progress to an AAA of 30 mm or greater within 5 years, none developed a clinically relevant AAA after 10 years of follow-up. Based on the findings of the present report, the follow-up strategy could be further specified; stratification of follow-up may already be possible at the time of initial screening. When an ASI of 13.0 or higher is used as a cut-off value at baseline screening, almost 30 per cent of men with an SAA are estimated to develop AAA at least 55 mm at 10.5 years. With that threshold, only about 50 per cent of all screening-detected SAAs would require a follow-up scan at 5 years, enabling identification of more than 90 per cent of those at risk of later becoming an AAA with a diameter of at least 55 mm.

Previous studies^14,19^ have indicated that aortic size adjusted for BSA is a better measurement than a fixed aortic diameter, which is common in screening programmes. Jones *et al*.^14^ reported only a modest difference in AAA prevalence in women compared with men when these factors were taken into consideration. A recent Swedish screening study^20^ reported an increased AAA prevalence from 1.5 to 1.9 per cent when aortic diameter was related to BSA compared with use of a fixed aortic threshold diameter of 30 mm in 65-year-old men.

Previous studies^15,21^ have shown an association between ASI and adverse AAA events and other cardiovascular events. Others^15^ reported that aortic diameter correlated to height (AHI) was independently associated with AAA events; this was also confirmed in the present study, with a five-fold risk of SAA later developing into an AAA of at least 55 mm with an AHI of 14.6 mm/m or greater.

Not surprisingly, for every millimetre increase in baseline aortic diameter, the incidence of AAA at least 55 mm escalated over time. The cumulative incidence of developing an AAA of 55 mm or larger was only 1.0 per cent in men with a 25-mm aortic diameter at baseline screening, whereas 33 per cent of those with a baseline aortic diameter of 29 mm were estimated to develop clinically relevant AAAs at 10.5 years (*Fig. 3d*). Based on these results, the value of offering further surveillance to men with an aortic diameter smaller than 26 mm can be questioned. It was suggested in previous studies^22,23^ from the Gloucestershire screening programme that an aortic diameter below 26 mm in 65-year-old men identified by screening does not need to be followed because these are unlikely to progress to clinically relevant AAAs. When an SAA diameter of at least 26 mm is used as cut-off value for further surveillance, the cumulative incidence of developing an AAA of at least 55 mm is about 20 per cent within 10 years. With that threshold, almost 75 per cent of all screening-detected SAAs would need to be followed up. By comparison, for men with an ASI of 13.0 mm/m^2^ or more, almost 30 per cent would eventually progress to an AAA of at least 55 mm, and only about half of those with an SAA at baseline screening would need follow-up. Using a combination of ASI 13.0 mm/m^2^ or more and SAA diameter of 26 mm or greater to predict progression of SAA to AAA at least 55 mm, only about 41 per cent of those with an SAA at baseline would eventually need follow-up. This proportion represents 0.8 per cent of all men screened. Based on the present results and findings from other studies^14,15,19,20^, a fixed aortic diameter of 30 mm, which is widely used in clinical practice, including screening programmes, entails a considerable risk of false-negative results. This is particularly obvious in women owing to their smaller mean aortic diameter^14,19^. The analysis of data on SAAs clearly shows that a considerable proportion of men with an aortic diameter of 25 mm or more are at risk of developing a clinically relevant AAA over time. The present data suggest that ASI could be a reliable and generalizable predictor for classifying these individuals with SAA who are at risk of later developing clinically relevant AAAs. However, these findings need to be validated in other cohorts.

Other suggested definitions relate the infrarenal aortic diameter to the adjacent segment of normal aorta^16,17^. When using these methods/definitions, the adjacent ‘normal’ aortic diameter must be measured, which could be challenging with ultrasonography^16,17^. Unexpectedly, in the present study, there was no statistically significant difference when the relative ratio and the relative difference between the infrarenal and adjacent normal aorta segment were investigated regarding development of AAA at least 55 mm (*Fig. 4*). In a previous screening study^24^, a lower prevalence was reported with use of infrarenal/suprarenal aortic diameter ratio compared with a fixed aortic diameter measurement, and it was even noted that some larger aortic diameters exceeding 40 mm were considered as non-aneurysmal aortas. According to these definitions in the present study, 82 and 89 per cent of those with SAAs would be classified as having an AAA at baseline screening. However, there was no association with progression to AAA at least 55 mm.

Ultrasound imaging is the most common and practical method for AAA screening, with high sensitivity and specificity for detecting AAAs. However, despite recent advances in ultrasound technology, measurement errors can result from intraobserver and interobserver errors^25^, and variation of up to 5 mm has been reported^26,27^. Nevertheless, the observed clear association between the diameter obtained and progression suggests that it is likely to be a minor problem in practice. The measured aortic diameter significantly depends on the measurement method used^9^. The inner-to-inner (ITI) technique used in the UK screening programme provides measurements that can be as much as 4 mm smaller than the outer-to-outer (OTO) measurement, and 2 mm smaller than the measurement obtained using the LELE technique currently employed in Sweden^8,28,29^. The choice of measurement method therefore has a major impact on the natural course of borderline aneurysms; the use of ITI measurement results in more SAAs being at risk of later developing into clinically significant AAAs, whereas the problem is less with the OTO technique than reported in this study. Other limitations of ultrasonography are suboptimal imaging and inaccurate measurements owing to bowel gas and obesity, and difficulty visualizing the suprarenal or proximal normal aortic segment^11,27,30^. This could to some extent be a limitation when using the relative aortic diameter methods or definitions, which require an ultrasound measurement of the adjacent aortic segment. The present results suggest that relative aortic diameter quotient and/or difference are not reliable predictors of later development of AAA at least 55 mm in AAA screening or surveillance. The disadvantage of these methods is uncertainty regarding whether the proximal adjacent aortic segment is normal or not. The patient may have a suprarenal or pararenal AAA as only a small segment of the suprarenal aorta can be visualized by ultrasound imaging. However, if information on height and weight is available, ASI and AHI can easily be calculated, and an expected normal aortic diameter could be estimated.

The association between enlarged aortic diameter and traditional risk factors, such as smoking, cardiovascular disease, hypertension, and hyperlipidaemia^31,32^, as well as that between AAA and increased all-cause mortality, has been well documented in several previous population-based studies^33–35^. In the present study, having a first-degree relative with AAA and smoking duration were found to be independent risk factors for progression to AAA at least 55 mm.

An important limitation of this study is that the time-to-event analysis is misleading to some extent as the event is based on the surveillance interval, rather than a continuous event. However, with larger AAAs, follow-up intervals are more frequent. Moreover, with a median duration of follow-up of 6.6 years for the whole cohort, only a small proportion had developed an AAA of 55 mm or larger so the results must be interpreted with caution. The data on length, weight, smoking habits, heredity, and co-morbidities were self-reported as part of a questionnaire filled out by individuals during screening, and may be at risk of overestimation or underestimation based on patients’ understanding. However, with the clear correlation observed between BSA and baseline infrarenal aortic diameter, this limitation appears to be less important in the present study.

General ultrasound screening of 65-year-old men for AAA has proved to be highly cost-effective^1,2^, even with a decreasing prevalence of the disease. This conclusion was confirmed in the Swedish screening programme, in which it was observed that about 40 per cent of all men with a screening-detected AAA would need surgical treatment over a lifespan^2^. Given a prevalence of less than 1.5 per cent, 0.6 per cent of the whole screening population could benefit from screening and follow-up surveillance. In the subgroup of men with SAAs, much more than 0.6 per cent of those offered follow-up could benefit from surveillance. Offering a population of 65-year-old men with SAAs (re)screening after 5 years would generate a significantly higher yield, with an estimated proportion of nearly 30 per cent developing clinically relevant AAAs after 10 years. A follow-up policy based on morphological characteristics in this cohort with screening-detected SAAs has the potential to further improve the efficiency of screening programmes and is likely to be cost-effective. However, a formal evaluation of cost-effectiveness in this subgroup is warranted.

The knowledge that one has an AAA, but that it will not be treated until it grows larger, could lead to possible psychosocial harm and decreased quality of life. This could be particularly relevant for men with an SAA at the age of 65 years, who may require surveillance for up to 10 years or more before they eventually develop a clinically relevant AAA; a considerable proportion will never meet the criteria for elective repair owing to old age and co-morbidities. Therefore, many authors have questioned whether individuals with SAAs benefit from follow-up surveillance^23,32,36^. However, based on findings from the present analysis and other studies^6,7^, a considerable number were considered eligible for repair. Furthermore, it could be problematic to defend rupture and AAA-related death after being declared healthy from aneurysm disease with an SAA at the 65-year screening. There is, however, a lack in the literature regarding the benefits and harms in this subgroup; this is an area of interest that needs to be investigated.

## Supplementary Material

znad030_Supplementary_Data

## Data Availability

The authors state that all data in the present study are available.
