# Supplementary material for: Morphological factors associated with progression of subaneurysmal aortas
Source: Br J Surg. 2023 Mar 4;110(4):489–97. doi: 10.1093/bjs/znad030 (PMC10364530; doi:10.1093/bjs/znad030)
Supplement: znad030_Supplementary_Data [file znad030_supplementary_data.docx]

**Morphological factors associated with progression of sub-aneurysmal aortas to clinically relevant abdominal aortic aneurysms**

Knut Thorbjørnsen, MD^1,2,3^, Sverker Svensjö, MD, PhD^1,4,5^, Kevin Mani, MD, Phd^1^, Anders Wanhainen, MD, PhD^1,6^

1) Department of Surgical Sciences, Section of Vascular Surgery, Uppsala University, Uppsala, Sweden

2) Centre for Research and Development, Uppsala University, Region Gävleborg, Sweden

3) Department of Surgery, Gävle County Hospital, Gävle, Sweden

4) Centre for Clinical Research, Uppsala University, Region Dalarna, Sweden

5) Department of Surgery, Falun County Hospital, Falun, Sweden

6) Department of Surgical and Perioperative Sciences, Surgery, Umeå University, Umeå, Sweden

**Corresponding author:** Knut Thorbjørnsen, MD, Centre for Research and Development, Uppsala University, Region Gävleborg, 80188 Gävle, Sweden, Phone: +46 26 15 42 89, Email: [knut.thorbjornsen@regiongavleborg.se](mailto:knut.thorbjornsen@regiongavleborg.se).

ORCID ID; 0000-0003-4404-5406

**Supplementary Materials - Index**

| **Figure S1a** | *pag.2* |
| --- | --- |
| Reciver operating characteristic (ROC) curve - aortic size index (ASI) |  |
| **Figure S1b** | *pag. 2* |
| Box plot and whisker plot – aortic size index (ASI) |  |
| **Figure S2a** | *pag.3* |
| Reciver operating characteristic (ROC) curve – aortic height indiex (AHI) |  |
| **Figure S2b** | *Pag.3* |
| Box plot and whisker plot – aortic height index (AHI) |  |
| **Figure S3a** | *pag. 4* |
| Reciver operating characteristic (ROC) curve – baseline (sub-aneurysmal) aortic diameter |  |
| **Figure S3b** | *pag. 4* |
| Box plot and whisker plot – baseline (sub-aneurysmal) aortic diameter |  |
| **Figure S4a** | *pag. 5* |
| Reciver operating characteristic (ROC) curve – relative aortic diameter quotient |  |
| **Figure S4b** | *pag.5* |
| Box plot and whisker plot – relative aortic diameter quotient |  |
| **Figure S5a** | *Pag.6* |
| Reciver operating characteristic (ROC) curve – relative aortic diameter difference |  |
| **Figure S5b** | *Pag.6* |
| Box plot and whisker plot – relative aortic diameter difference |  |
| **Figure S6** | *Pag.7* |
| Correlation matrixes |  |

**Figure S1a**

**
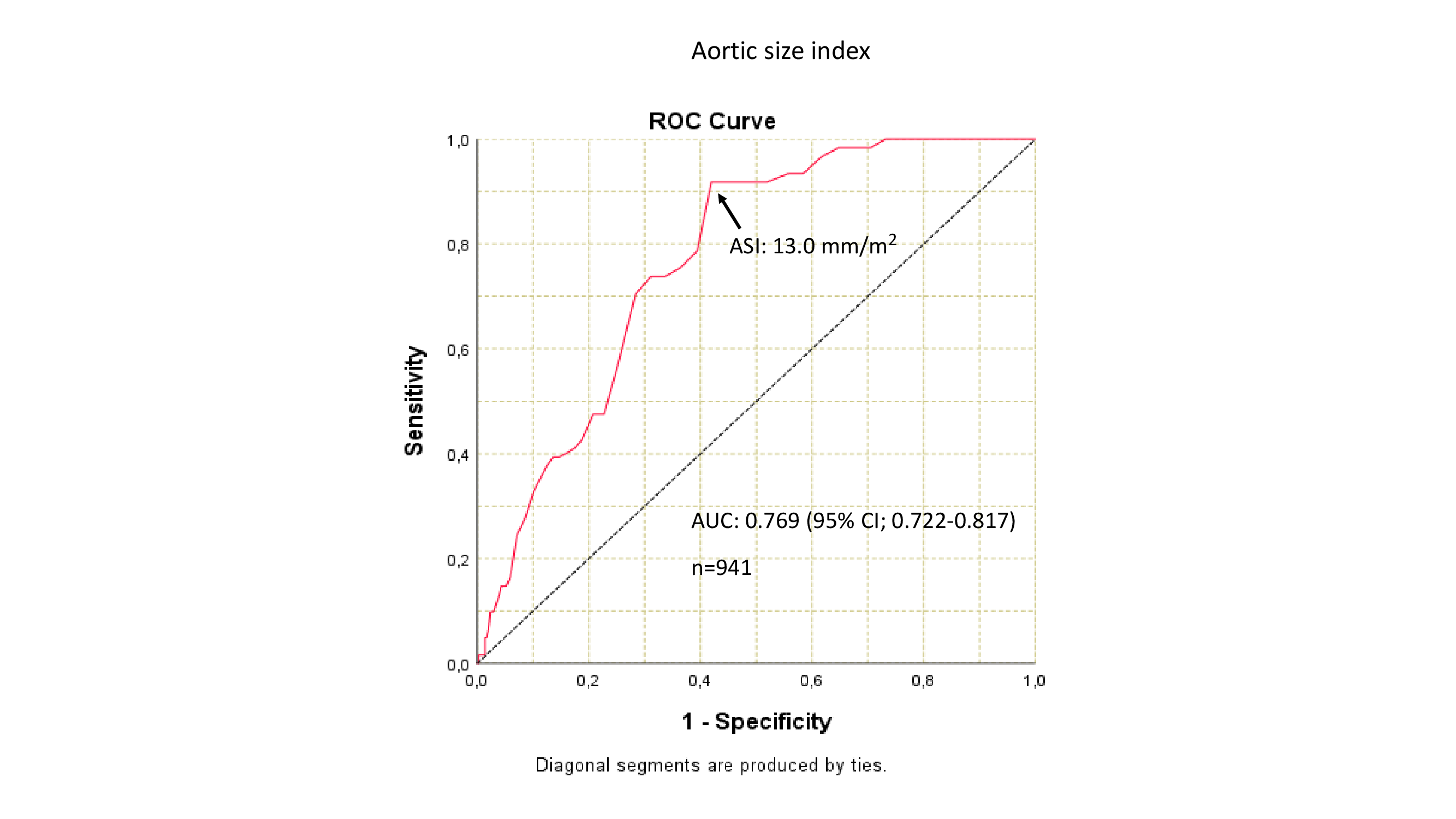
**

**Figure S1a:** Reciver-operating characteristic (ROC) curve analysis of the optimal cut-off value = 13.0 for aortic size index (ASI). The threshold for sensitivity was set to ≥ 90%.

**Figure S1b**

**
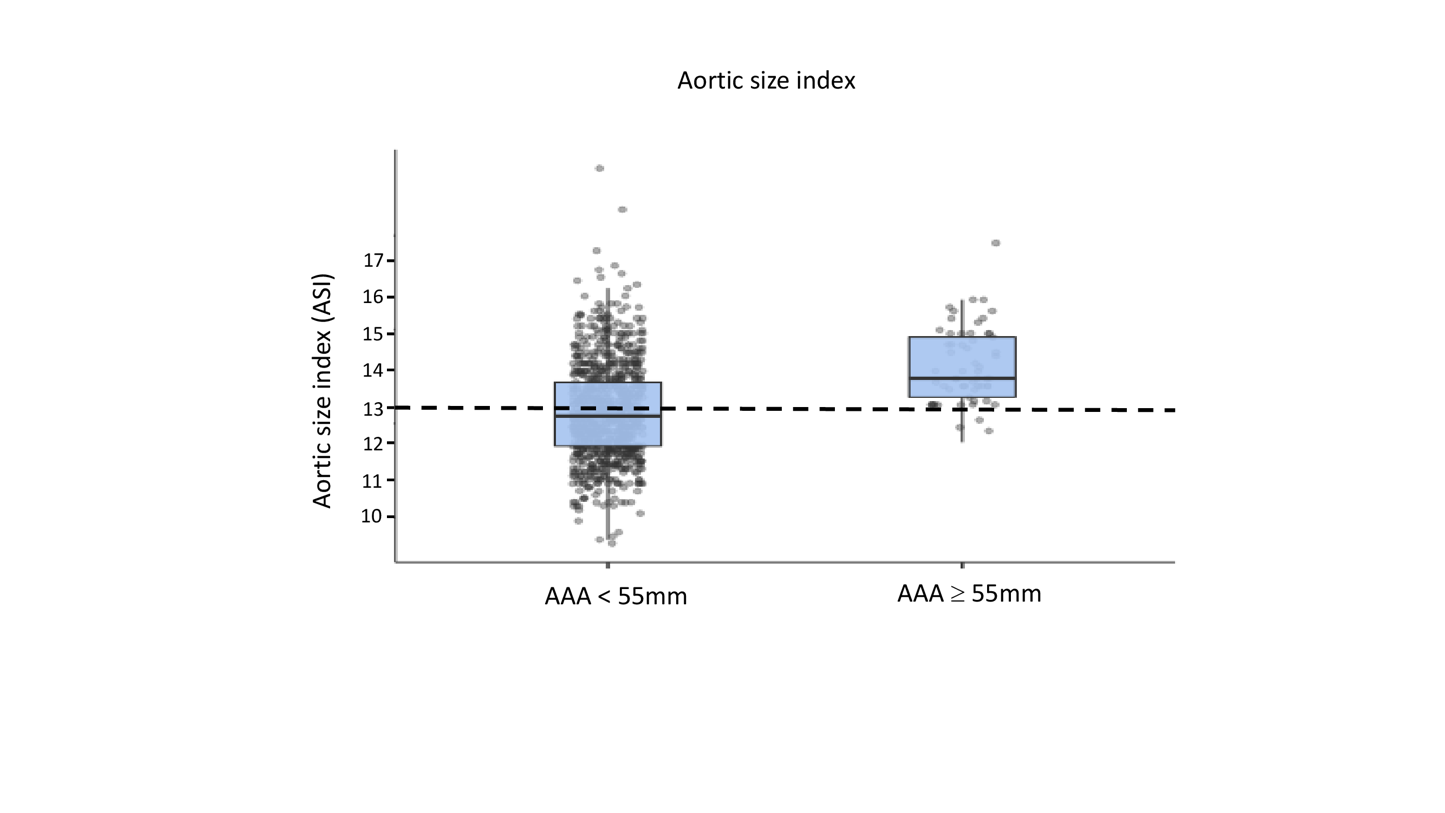
**

**Figure S1b:** Box plot and whisker plot of aortic size index (ASI) split by aortic diameter < 55 mm and ≥ 55 mm. The horizontal dotted line represent the optimal cut-off value of 13.0.

**Figure S2a**


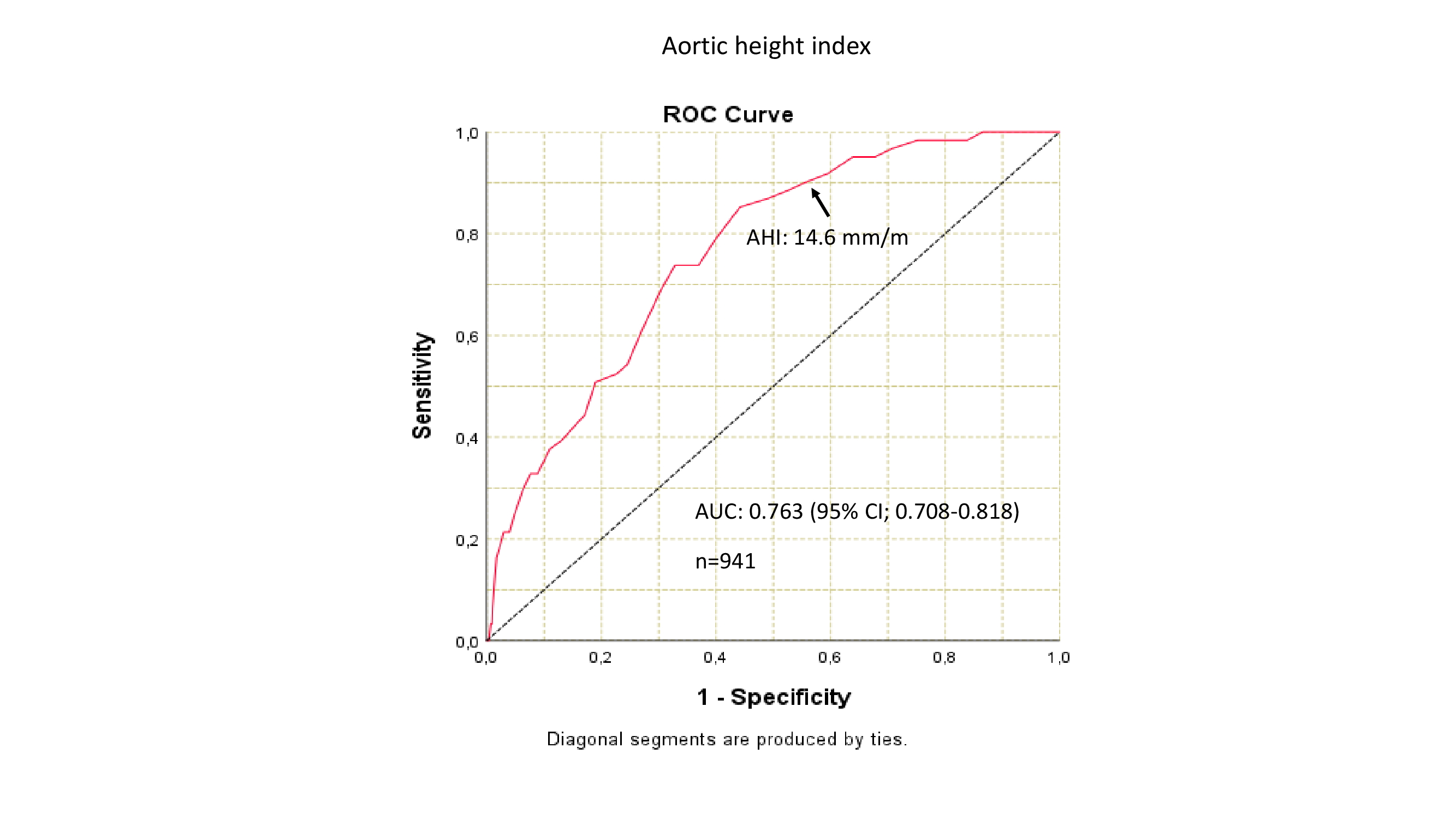


**Figure S2a:** Reciver-operating characteristic (ROC) curve analysis of the optimal cut-off value = 14.6 for aortic height index (AHI). The threshold for sensitivity was set to ≥ 90%.

**Figure S2b**


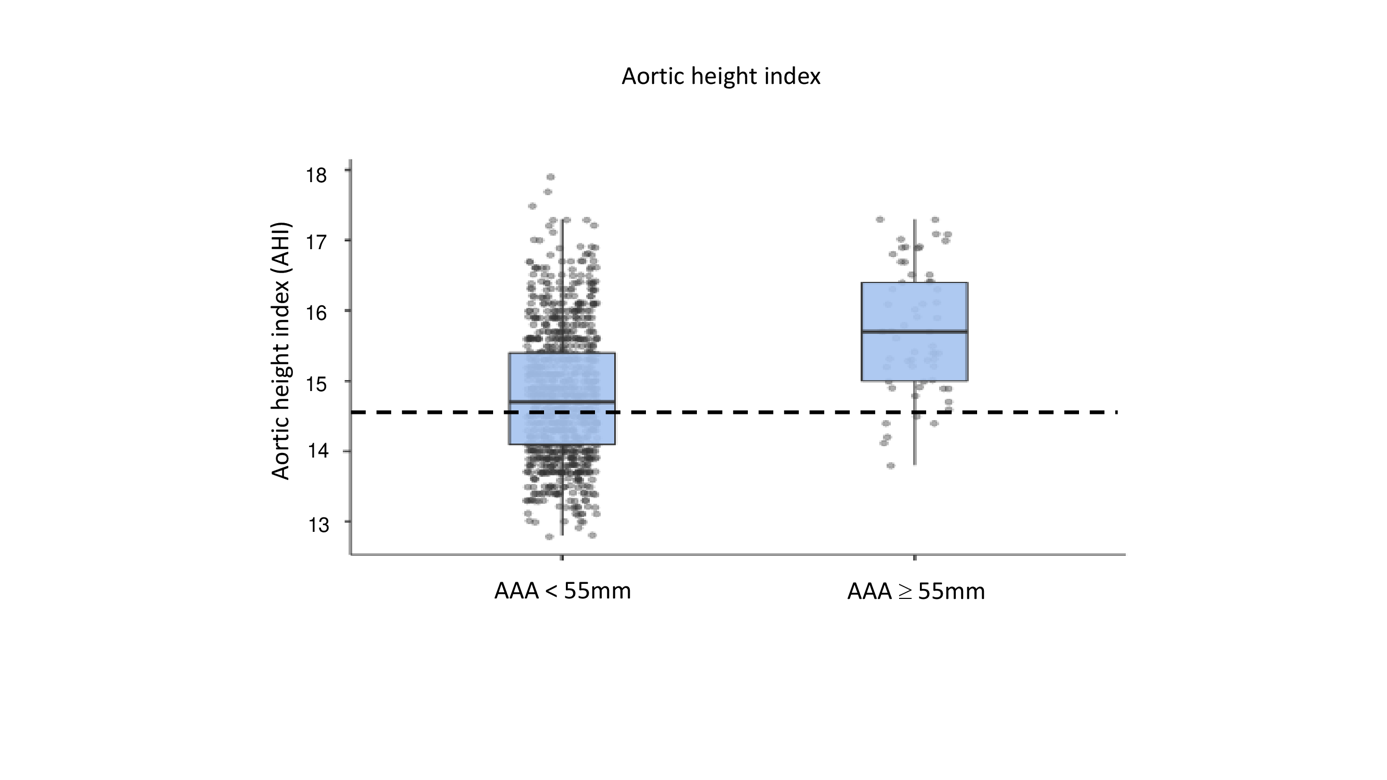
 **Figure S2b:** Box plot and whisker plot of aortic height index (AHI) split by aortic diameter < 55 mm and ≥ 55 mm. The horizontal dotted line represent the optimal cut-off value of 14.6.

**Figure S3a**


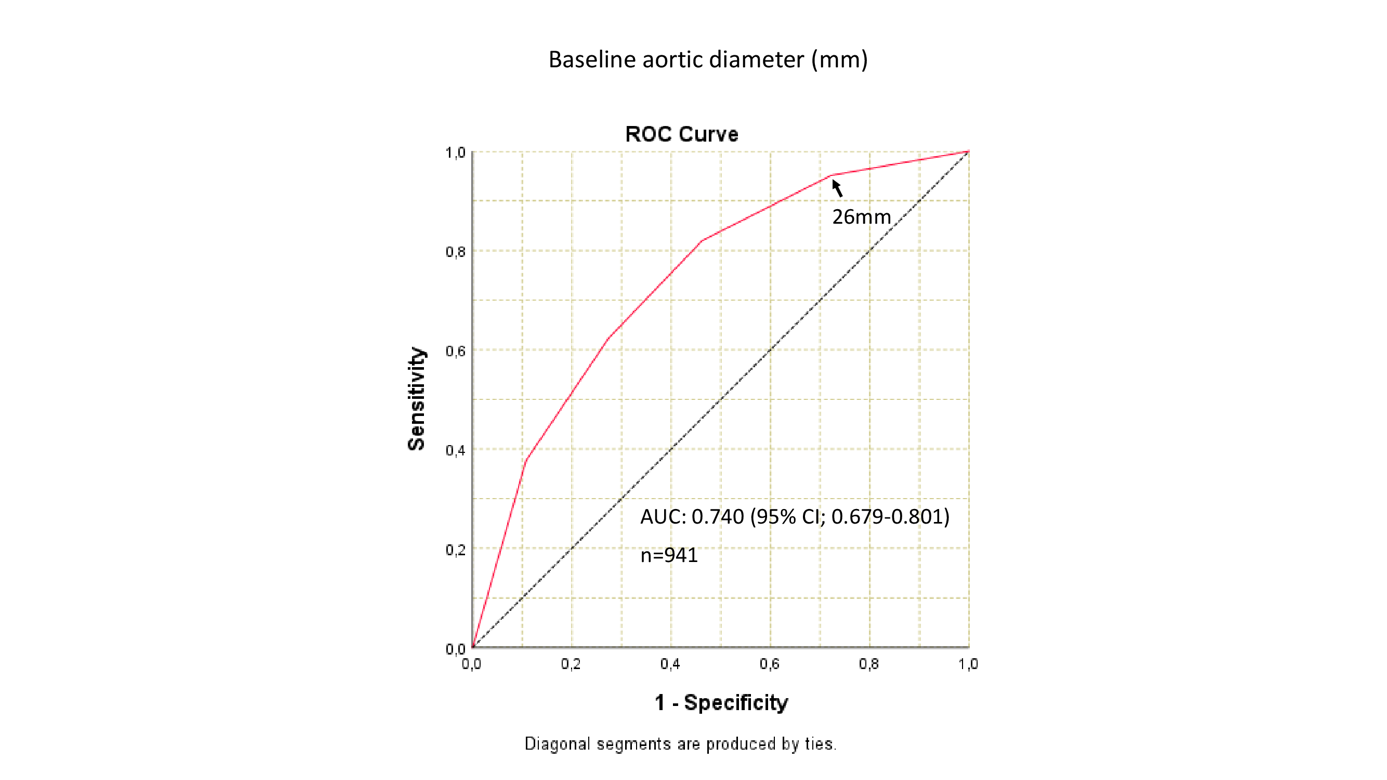


**Figure S3a:** Reciver-operating characteristic (ROC) curve analysis of the optimal cut-off value = 26 mm for baseline (sub-aneurysmal) aortic diameter. The threshold for sensitivity was set to ≥ 90%.

**Figure S3b**


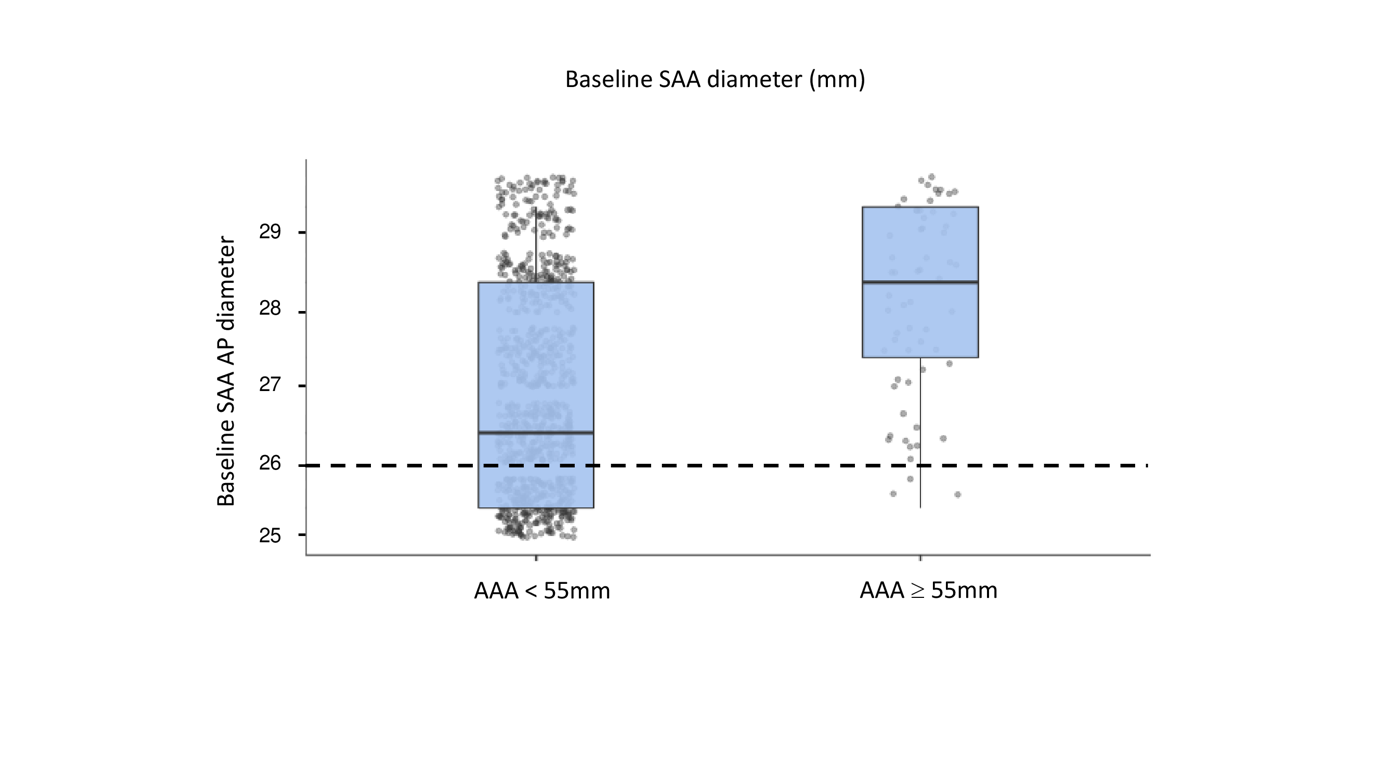


**Figure S3b:** Box plot and whisker plot of baseline (sub-aneurysmal) aortic diameter split by aortic diameter < 55 mm and ≥ 55 mm. The horizontal dotted line represent the optimal cut-off value of 26 mm.

**Figure S4a**

**
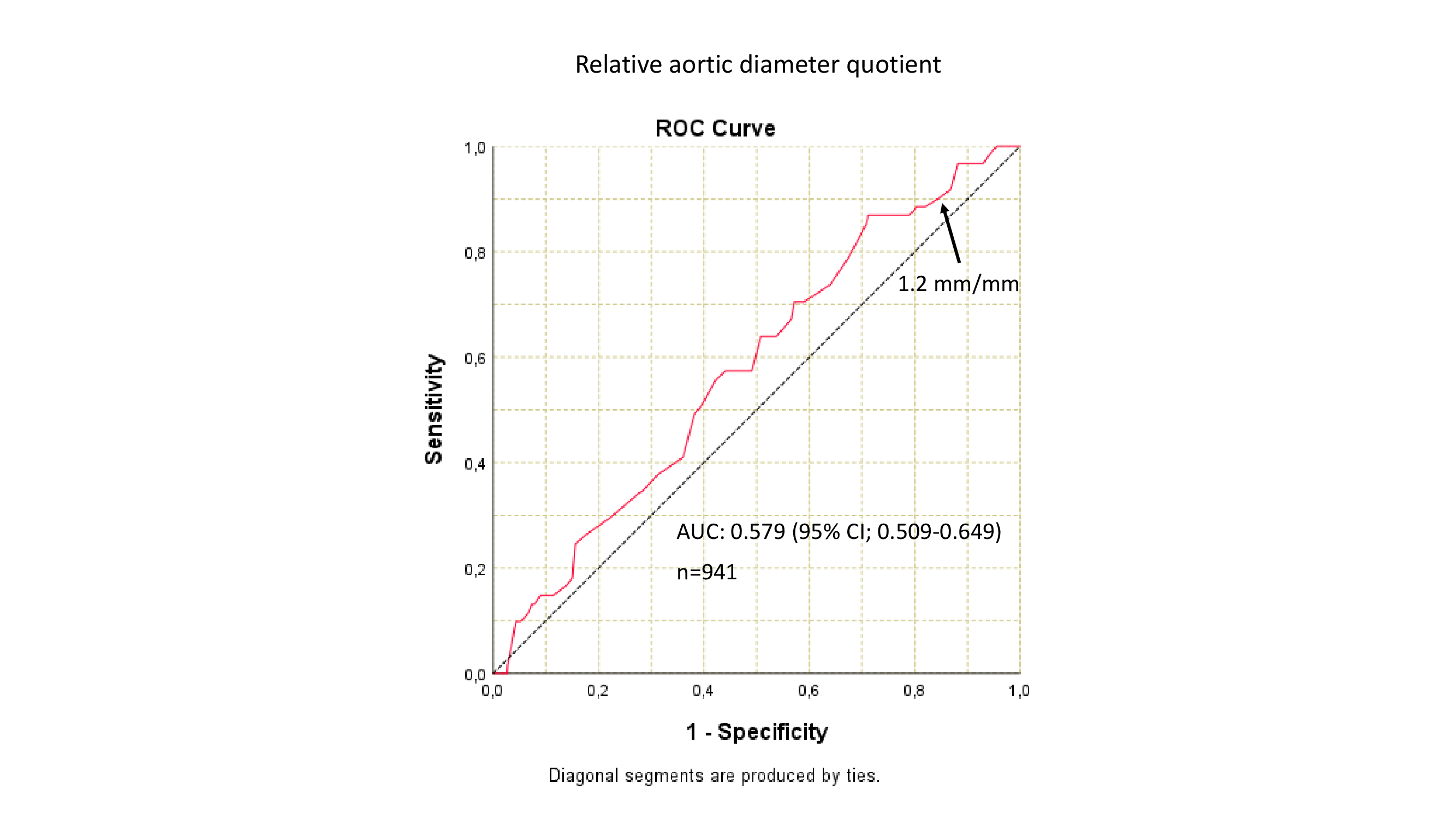
**

**Figure S4a:** Reciver-operating characteristic (ROC) curve analysis of the optimal cut-off value = 12.0 for relative aortic diameter quotient. The threshold for sensitivity was set to ≥ 90%.

**Figure S4b**


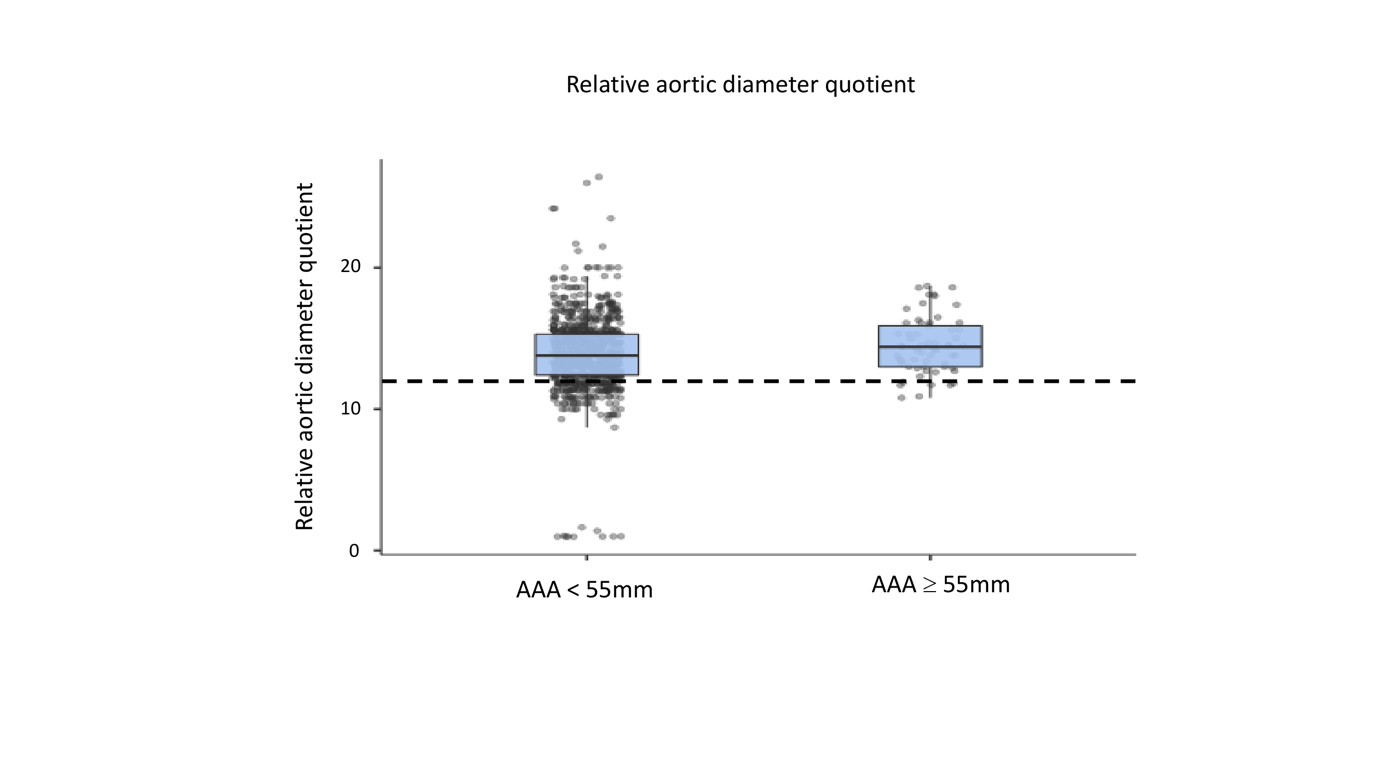
**Figure S4b:** Box plot and whisker plot of relative aortic diameter quorient split by aortic diameter < 55 mm and ≥ 55 mm. The horizontal dotted line represent the optimal cut-off value of 12.0.

**Figure S5a**


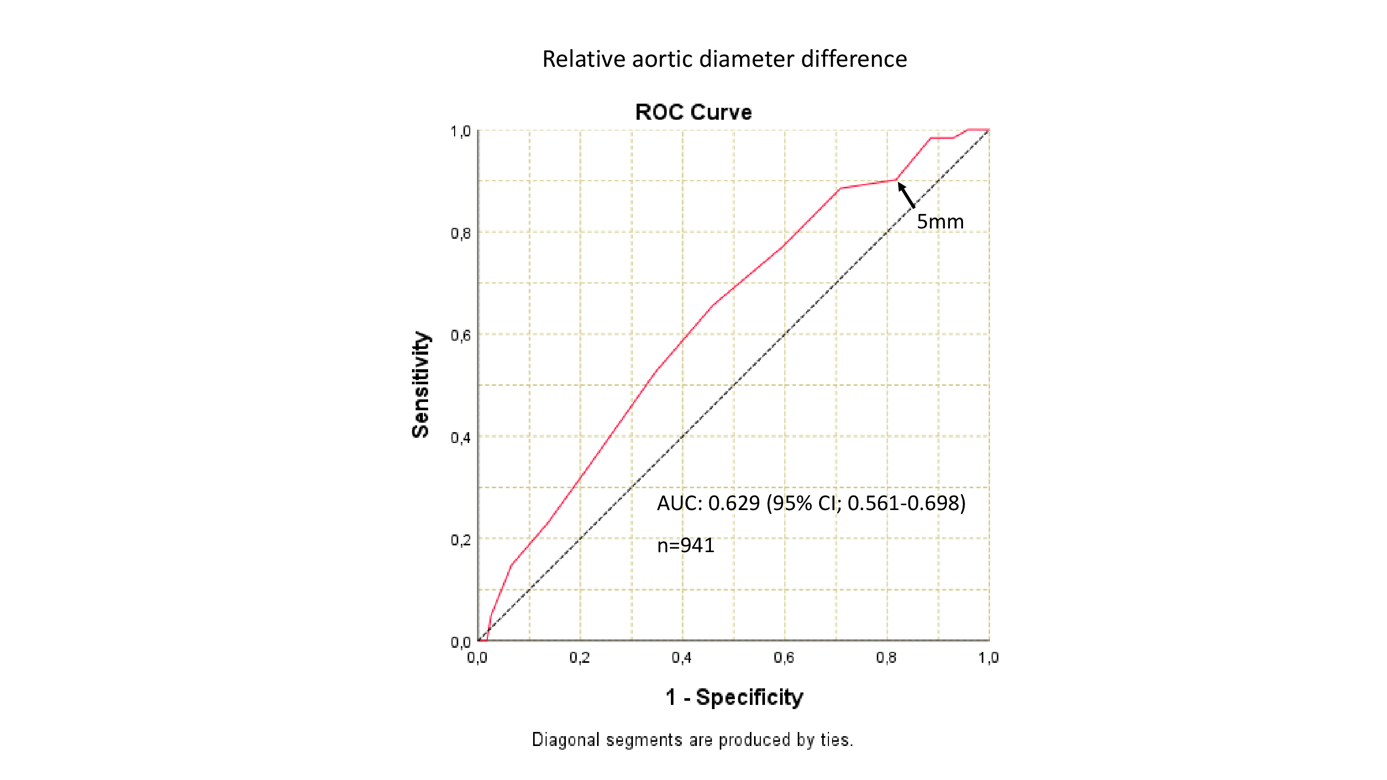
**Figure S5a:** Reciver-operating characteristic (ROC) curve analysis of the optimal cut-off value = 5 mm for relative aortic diameter difference. The threshold for sensitivity was set to ≥ 90%.

**Figure S5b**


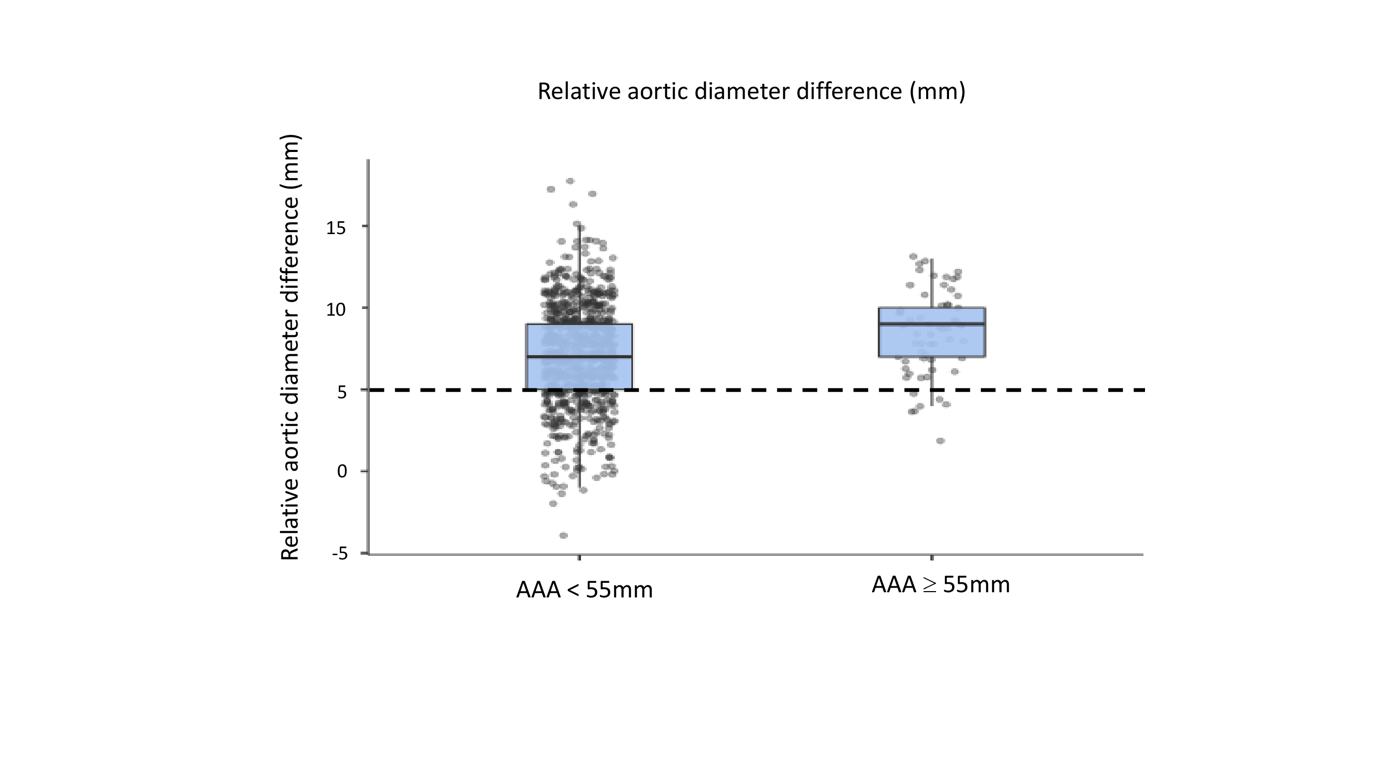


**Figure S5b:** Box plot and whisker plot of relative aortic diameter difference split by aortic diameter < 55 mm and ≥ 55 mm. The horizontal dotted line represent the optimal cut-off value of 5 mm.

**Figure S6**

**
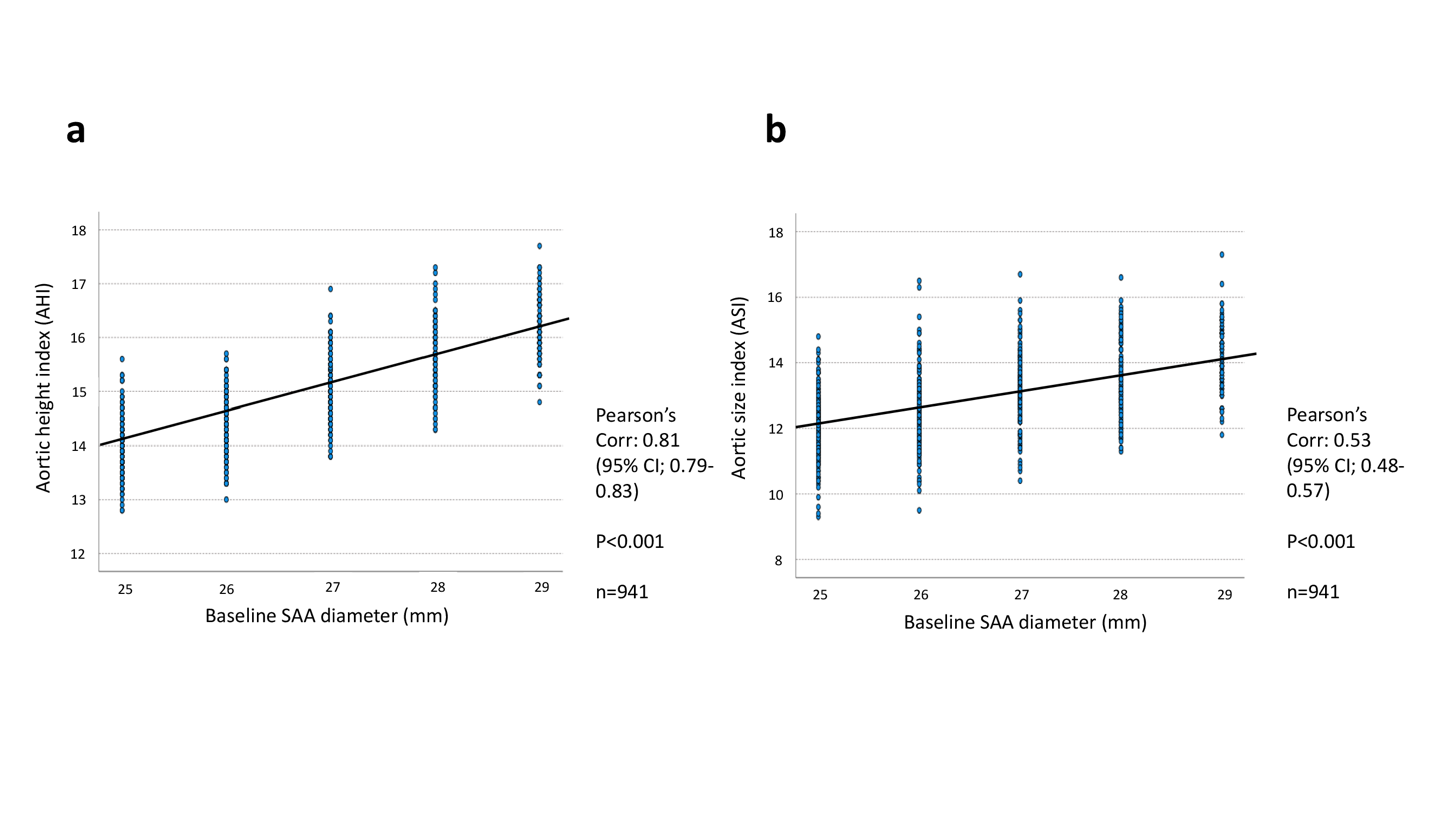
 Figure S6:** a) Correlation matrix between baseline (sub-aneurysmal) aortic diameter (mm) and aortic height index (AHI), b) Correlation matrix between baseline (sub-aneurysmal) aortic diameter (mm) and aortic size index (ASI).
